# A Rare Case of Giant Cystic Adenomatoid Tumor of the Uterus With Literature Review

**DOI:** 10.1155/2024/7791245

**Published:** 2024-10-23

**Authors:** Pranav S. Renavikar, Lina Adwer, David G. Wagner, Subodh M. Lele

**Affiliations:** ^1^Department of Pathology, Microbiology, and Immunology, University of Nebraska Medical Center, Omaha, Nebraska 68198, USA; ^2^College of Medicine, University of Nebraska Medical Center, Omaha, Nebraska, USA

**Keywords:** cystic adenomatoid tumor, leiomyoma, mesothelioma, uterus

## Abstract

Adenomatoid tumors are rare benign neoplasms arising from mesothelial cells, commonly found in the female genital system, particularly the uterus and fallopian tubes. The giant cystic variant of adenomatoid tumor is exceptionally rare and can cause massive growth mimicking malignant gynecological conditions. Histology and immunohistochemistry play a crucial role in confirming the diagnosis, with markers such as calretinin, D2-40, CK7, BAP1, ER, and WT1 proving useful. A 51-year-old female with a history of breast cancer presented with pelvic pressure and vague pain. Imaging revealed an enlarged uterus with multiple heterogeneously enhancing masses and a predominantly cystic mass arising from the fundus, all believed to be leiomyomas. Surgical exploration and subsequent pathologic examination identified the cystic tumor as cystic adenomatoid tumor coexisting with leiomyomas, adenomyosis, and abdominal endometriosis. Diagnosing cystic adenomatoid tumor presents challenges, especially in patients with complex gynecologic histories. Cystic adenomatoid tumors typically have a favorable prognosis following surgical intervention. This case demonstrates one of the few reports of a giant cystic adenomatoid tumor (11.5 cm) and highlights diagnostic mimics. As these tumors are typically small and often seen only microscopically, the large size can confuse the pathologist who may be unaware of this feature leading to a misdiagnosis.

## 1. Introduction

Adenomatoid tumors are specialized, nonrecurring, benign neoplasms derived from mesothelial cells [1–3]. These tumors are predominantly found in the genital systems of both males and females and rarely in extragenital sites like the adrenal gland and peritoneum. However, uncommon extensive involvement of the superficial myometrium, or extension to the endometrium or serosa, is noted [1, 3, 4].

In males, the most common site is the epididymis [2–4]. In females, they are primarily found in the uterus with an incidence of less than 1% to 5% [1–4]. The exact incidence of adenomatoid tumors is not well-established due to their incidental nature in hysterectomy specimens. Cases are common in women who are 25–65 years of age, with the most frequent clinical presentation being menorrhagia [2].

Four basic histological types of adenomatoid tumors are identified: adenoid, angiomatoid, solid, and cystic, with the cystic variant being the rarest. It is common to find an overlap of two or more histologic types in a single tumor [2]. Radiologically, these tumors can appear as small solid to cystic masses. Grossly, adenomatoid tumors most commonly appear as small, solid, well-circumscribed, firm, gray-tan to white masses, resembling leiomyomas [1, 4]. They can also appear as large cystic adenomatoid tumors that morphologically resemble multicystic mesotheliomas [2, 5].

Our case highlights the rare giant cystic presentation of adenomatoid tumor of the uterus, where histopathology and immunohistochemistry played a crucial role in the diagnosis. Differential diagnoses for cystic adenomatoid tumors include leiomyoma with cystic degeneration, cystic endometriosis, vascular tumors, and adenocarcinoma. They must also be distinguished from mesothelial lesions like well-differentiated papillary mesothelioma and benign multicystic mesothelioma [4].

## 2. Case Description

**Figure 1:**
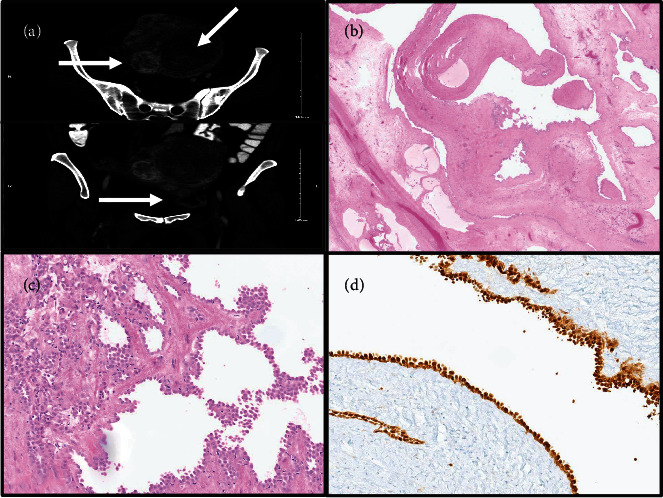
Radiologic and pathologic assessment of giant cystic adenomatoid tumor. CT scan showing the large uterine masses with intramural and subserosal locations, along with the cystic mass ((a) axial and coronal planes, arrows). Microscopy of the uterine mass showing cystic spaces lined by mesothelial cells with focal areas of clustering in a background of fibrous and edematous stroma. No atypia, necrosis, or mitoses were seen ((b, c) H&E staining, 2x and 20x, respectively). Immunohistochemical stain for calretinin is positive in the lesional cells ((d) 20x).

The patient is a 51-year-old premenopausal female with a history of breast cancer diagnosed one year prior to current presentation, pathologically consistent with invasive ductal carcinoma Nottingham Grade 2/3, and treated with margin negative lumpectomy and sentinel lymph node biopsy (pathologic stage: pT1c pN0), followed by adjuvant radiation. Her primary symptoms included pelvic pressure and vague pelvic pain. No changes in bowel or bladder habits, abnormal vaginal discharge, irregularities in menstrual cycles, or heavy bleeding were reported. Family history was significant for breast and ovarian cancers in multiple maternal family members. However, the patient's BRCA1 and BRCA2 gene mutation status was negative. Physical examination revealed no abnormality in the urethra, urinary bladder, cervix, vagina, and rectum. On bimanual examination, the lower uterine segment was enlarged, smooth, and mobile, with no demonstrable adnexal masses. Imaging studies (CT scan) revealed that the uterus was enlarged, measuring 13.5 × 10.5 cm in axial dimension and 19.5 cm craniocaudal. It contained numerous, mostly intramural heterogeneous-enhancing masses, consistent with leiomyomas. The lesion of interest was predominantly cystic, radiologically believed to be a subserosal leiomyoma arising in the uterine fundus and spanning the left pelvis (Figure 1(a)). The right ovary contained simple cysts, while the left ovary was not visualized on imaging studies.

Preoperatively, the uterine masses were thought to represent leiomyomas, while the surgical differential for the cystic mass included subserosal leiomyoma with cystic change and ovarian mucinous lesions like cystadenoma, borderline tumor, or carcinoma. The patient underwent open exploratory laparotomy with total abdominal hysterectomy, bilateral salpingo-oophorectomy, and resection of the cystic mass. Intraoperatively, the uterus was enlarged to approximately 20 cm and noted to have multiple fibroids. A large mass attached to the uterine fundus (approximately 10 cm) and consisting of numerous small blebs and cysts of yellow-brown gelatinous material were removed. Intraoperative frozen section pathology favored benign multicystic lesion. Bilateral ovaries, fallopian tubes, and appendix had no significant abnormality.

Pathological examination of the resected uterine mass revealed a benign cystic adenomatoid tumor. On gross examination, the mass was tan-pink, multilocular cystic, and contained gelatinous fluid (11.5 cm in greatest dimension). Microscopically, cystic branching spaces lined by mesothelial cells with focal areas of clustering were seen in a background of fibrous and edematous stroma (Figures 1(b) and 1(c)). No atypia, necrosis, or mitoses were seen. Minor areas of conventional gland-like and solid morphology were also noted in the myometrium. Additional findings included benign proliferative endometrium, leiomyomas and adenomyosis in the myometrium, ovarian follicular and serous cysts, and abdominal wall endometriosis. Immunostaining results revealed that the cystic lesional cells were positive for calretinin (Figure 1(d)), D2-40 (patchy), CK7, BAP1, and WT1, and negative for PAX8, ER, ERG, CD31, CDX2, SATB2, and CK20.

Upon a follow-up duration of 48 months with regular mammograms and CT scans, there was no evidence of recurrent breast cancer or cystic adenomatoid tumor, respectively.

## 3. Discussion

Despite cystic adenomatoid tumor's benign nature, its clinical presentation can mimic a variety of gynecological conditions. Thus, comprehensive histopathological and immunohistochemical analyses are crucial for definitive diagnosis [1–5]. The differential diagnosis of such tumors necessitates considering several benign conditions like leiomyomas, adenomyosis, and ovarian mucinous cystadenoma, among others. Despite the focus on benign conditions, the possibility of malignant entities cannot be ignored, especially given the patient's breast cancer history. Conditions such as metastatic carcinomas (including signet ring cell carcinoma), ovarian mucinous carcinoma, and malignant mesothelioma must be considered in the differential diagnosis [4]. The cystic mass presented as an apparent uterine mass in a background of radiologically described multiple leiomyomas. Thus, the cystic mass was perceived as being a subserosal leiomyoma. The size of >10 cm and extension into the left pelvis warranted the inclusion of other differentials like cystic endometriosis/endosalpingiosis, ovarian mucinous tumors (cystadenoma, borderline tumor, or adenocarcinoma), metastatic gastrointestinal carcinoma, other mesothelial lesions (well-differentiated papillary mesothelioma, benign multicystic mesothelioma, and malignant mesothelioma), and rare vascular tumors like lymphangioma. Due to the patient's age, symptoms, large size of the mass, and potential for malignancy, an operative management was preferred. Intraoperatively, the cystic mass was attached to the uterine fundus, while the ovaries and tubes did not show significant lesions. The appendix was unremarkable, and no other peritoneal deposits were seen. Intraoperative frozen section assessment revealed a benign multicystic lesion. Thus, the differentials of ovarian mucinous tumors, metastatic gastrointestinal malignancy, and malignant mesothelioma were potentially ruled out. Upon pathologic examination, the cystic spaces were lined by benign mesothelial cells in a background of fibrous and edematous stroma, with scattered minor areas of conventional gland-like and solid morphology of adenomatoid tumor in the myometrium. Significant papillary morphology with myxoid change, psammoma bodies, and endometrial-type stroma were not seen in the lesion. Immunostains confirmed the benign mesothelial cell phenotype. The gross and histologic findings ruled out well-differentiated papillary mesothelioma, cystic endometriosis/endosalpingiosis, and lymphangioma.

The pathological and immunohistochemical findings in our case, including positive staining for calretinin, D2-40, CK7, BAP1, and WT1, and negativity for various other markers, confirmed the diagnosis of a benign cystic adenomatoid tumor and ruled out metastatic disease. In terms of size and type variations, adenomatoid tumors of the uterus display a broad spectrum. A study by Nogales et al. categorizes uterine adenomatoid tumors into two distinct macroscopic patterns: small, solid tumors (common) and large, cystic ones (rare). Most adenomatoid tumors were small, solid tumors found incidentally in hysterectomies, while only a few cases were large and cystic (similar to our case), classified as “giant cystic adenomatoid tumors” due to their substantial size and distinctive appearance. The rare large cystic tumors in their study ranged from 7 to 10 cm in size (four cases) and showed multicystic masses with spongy and honeycomb appearance. Two of their cases protruded from the serosal surface due to dilatation, while one case (like ours) showed exophytic grape-like lesion in continuity with the serosal surface [3]. While they can be confused with leiomyoma or adnexal masses due to their cystic nature on ultrasound [3], other studies also document helpful radiologic assessment wherein one patient's MRI findings assisted in the diagnosis of cystic adenomatoid tumor initially suspected to be a large leiomyoma with cystic degeneration, and another patient with suspected ruptured ovarian mucinous cystadenoma was later identified to have an adenomatoid tumor, as the normal-sized ovary was not visible next to the tumor [5]. Our case describes one of the largest reported sizes for cystic adenomatoid tumor (11.5 cm), corroborates prior knowledge regarding this entity, creates a rationale for considering differential diagnoses, provides a detailed intraoperative description of findings with frozen section assessment (data on which are limited), and importantly makes aware of its benign nature on long-term follow-up.

Further, the mesothelial cells of adenomatoid tumors typically lack estrogen or progesterone receptors, like normal peritoneum and other mesotheliomas. Immunohistochemically, these tumors show strong positivity for low molecular-weight cytokeratin and mesothelial markers like calretinin and HBME-1 but not for EMA or thrombomodulin, further supporting their mesothelial origin [3]. Adenomatoid tumors express keratins, calretinin, WT1, and other mesothelial markers but are negative for CEA, factor VIII, and CD31. They also show retained BAP1 expression unlike malignant mesotheliomas. Evidence suggests a neoplastic nature for adenomatoid tumors, including monoclonality. Recent studies show TRAF7 mutations, indicating a potential link with immunosuppression [4]. The pathogenesis of adenomatoid tumors is not fully understood, with theories suggesting an origin from the uterine peritoneum or a precursor of both mesothelial and muscle cells. Understanding the molecular biology of adenomatoid tumors, especially the role of TRAF7 mutations, has provided insights into their pathogenesis and potential links with other mesothelial diseases. However, further research is needed to understand the connection between inflammation and tumor development and the exact relationship with other mesothelial lesions [4].

Clinically, cystic adenomatoid tumors generally follow a benign course, leading to successful outcomes postsurgical intervention. This benign trajectory is critical to patient management and prognosis [1–5]. In conclusion, the case we present, with its complex gynecological history and other conditions like leiomyomas, endometriosis, and benign ovarian cysts, underscores the importance of comprehensive pathological examination. This case aligns with the characteristics of larger cystic adenomatoid tumors, reinforcing the spectrum of these tumors as documented in scientific literature and illustrating the continuum from smaller, solid forms to larger cystic ones [2, 3].

## Data Availability

The authors have nothing to report.
